# A Broad Review of Soybean Research on the Ongoing Race to Overcome Soybean Cyst Nematode

**DOI:** 10.3390/biology11020211

**Published:** 2022-01-28

**Authors:** Nour Nissan, Benjamin Mimee, Elroy R. Cober, Ashkan Golshani, Myron Smith, Bahram Samanfar

**Affiliations:** 1 Agriculture and Agri-Food Canada, Ottawa Research and Development Centre, Ottawa, ON K1Y 4X2, Canada; nournissan96@gmail.com (N.N.); elroy.cober@agr.gc.ca (E.R.C.); 2 Ottawa Institute of Systems Biology and Department of Biology, Carleton University, Ottawa, ON K1S 5B6, Canada; ashkan_golshani@carleton.ca (A.G.); myron.smith@carleton.ca (M.S.); 3 Agriculture and Agri-Food Canada, Saint-Jean-sur-Richelieu Research and Development Centre, Saint-Jean-sur-Richelieu, QC J3B 7B5, Canada; benjamin.mimee@agr.gc.ca

**Keywords:** soybean, soybean cyst nematode (SCN), disease control, pathogen management, omics

## Abstract

**Simple Summary:**

The cultivated soybean [*Glycine max* (L.) Merr.] is an economically important crop worldwide and is regularly used for protein and oil in human consumption, animal feed, industrial products and as an important element in sustainable agricultural management practices due to its nitrogen fixation capability. Soybean cyst nematode, *Heterodera glycines* Ichinohe, is a plant parasitic nematode which is an overwhelming pest of soybean on a global scale. So far, soybean growers are limited to the soybean cultivars that can be planted in infested fields due to the lack of resistant genes identified against this pathogen. In this paper, we review a broad range of approaches which have been utilized in the race to understand the plant’s response to this nematode and the mechanisms of resistance, as well as to shed light onto the areas that need to be further investigated. The purpose of this review is to summarize the information that breeders and molecular biologists can use to better understand the host–pathogen relationship in the hopes of overcoming this devastating nematode.

**Abstract:**

Plant pathogens greatly impact food security of the ever-growing human population. Breeding resistant crops is one of the most sustainable strategies to overcome the negative effects of these biotic stressors. In order to efficiently breed for resistant plants, the specific plant–pathogen interactions should be understood. Soybean is a short-day legume that is a staple in human food and animal feed due to its high nutritional content. Soybean cyst nematode (SCN) is a major soybean stressor infecting soybean worldwide including in China, Brazil, Argentina, USA and Canada. There are many Quantitative Trait Loci (QTLs) conferring resistance to SCN that have been identified; however, only two are widely used: *rhg1* and *Rhg4*. Overuse of cultivars containing these QTLs/genes can lead to SCN resistance breakdown, necessitating the use of additional strategies. In this manuscript, a literature review is conducted on research related to soybean resistance to SCN. The main goal is to provide a current understanding of the mechanisms of SCN resistance and list the areas of research that could be further explored.

## 1. Introduction

### 1.1. Soybean

Soybean [*Glycine max* (L.) Merr.] cultivation occupies more than 6% of the world’s arable land with an ever-increasing production area. Soybean seeds contain around 31–44% protein and 19–26% oil, making soybean an excellent staple for both human food and animal feed [1]. Using soybean for crop rotation is also an important tool in sustainable agriculture due to its nitrogen fixation capability [2].

Based on analysis of the cultivar Williams 82, soybean has a genome size of ~1.1 GB contained in 20 chromosomes, with ~89,500 protein coding transcripts annotated for ~55,600 gene loci [3]. The soybean genome is believed to have undergone two major duplication events 59 and 13 million years ago, as well as many chromosomal rearrangements, and rounds of diploidization, all contributing to diversification of the genome [4]. This makes understanding the soybean genome complicated, given that ~75% of genes are present as paralogs [4].

Across the diversity of areas that soybean is cultivated, the plant must deal with various abiotic stressors relating to excess water, drought, iron and other mineral deficiencies, daylength, hail, wind and cold weather conditions [5]. For example, soybean is a short-day plant grown in latitudes 35°S to 50°N and is subjected to photoperiod sensitivities; thus, challenges emerge when trying to expand its cultivation past those latitudes [6]. Soybean also deals with several biotic stressors. Among these is soybean cyst nematode *Heterodera glycines* Ichinohe, (SCN). SCN is one of the most devastating pathogens to soybean and is widely present in many areas around the world and is continuing to spread to regions of soybean production in North America [7]. Hence, it is important to study this pathogen and identify resistance genes/QTLs in soybean for use in effective management of the disease.

### 1.2. Soybean Cyst Nematode (SCN)

SCN is a plant parasitic nematode that causes major soybean yield loss (over $1.5 billion annually in the United States) [7]. It has a fully sequenced genome size of ~158 MB comprising of 9 chromosomes and about ~22,400 annotated gene models [8]. Once SCN is present in the soil, eradication is nearly impossible because some eggs contained within the nematode cysts can remain alive for up to ten years and the infective juveniles can be released from the cysts whenever conditions become favorable [9]. Depending on the environmental conditions, the life cycle of SCN can be completed over a 4-week period. The life stages are: egg stage, juvenile stages (J1-J4), and adult stages (female or male). The first two juvenile stages occur within the egg. Once the J1 is formed it molts within the eggshell into the J2. Triggered by environmental factors including the presence of a host plant root, the J2 will hatch from the egg and enter the root. The infective J2 stage enters the root of the host plant using their stylet and by secreting cell-wall-degrading enzymes (e.g., cellulases). The J2 then induces formation of a specialized metabolically active feeding site made up of multinucleate (syncytium) vascular tissue of the roots [9]. In an incompatible interaction (unsuccessful infection), the syncytium is still formed but degrades over time and is overcome by the surrounding cells, whereas in a compatible interaction the syncytium is maintained and expands [10]. The J2 become immobilized and continues to feed at the syncytium and then molt to the J3, J4 and then eventually into the adult stage. Male adults leave the soybean roots after several days of maturing, and no longer harm the soybean plant, while the females continue to feed and increase in size. Damage to soybean plants is largely due to the female feeding. The adult female swells and pushes through the root surface with only the head left in the root. She then releases a pheromone to attract males for mating. Mated females deposit some eggs within a gelatinous matrix at its posterior end, ready to hatch and infect more soybean within the same year. As many as 500 viable eggs remain within the female body that then encysts and dies. These eggs within the cyst can remain viable for years in the soil, until a new host is present and conditions are favorable for renewed infections [9].

**Figure 1:**
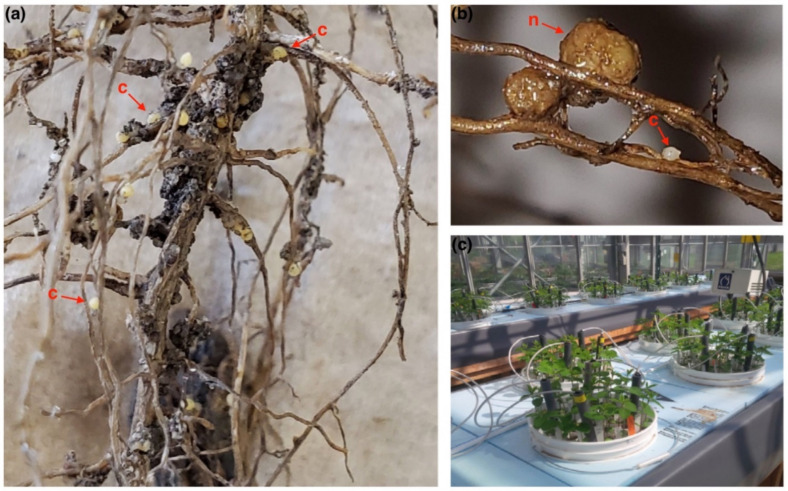
Soybean cyst nematode *Heterodera glycines* Ichinohe (SCN) infection on soybean roots and phenotyping facility. (**a**) SCN females shown on soybean roots; (**b**) A comparison between a nodule = n within the soybean root vs. a female nematode = c, highlighted with red arrows; (**c**) An SCN phenotyping facility at Agriculture and Agri-Food Canada, Saint-Jean-sur-Richelieu Research and Development Centre.

Symptoms of soybean infected with SCN include chlorosis of the leaves, darker and less developed roots, and stunting which leads to significant yield loss varying between 5–80%. Nodulation with nitrogen-fixing bacteria can also be reduced [9]. In general, there are significant economic losses caused by SCN. In a study from 1996–2016 across 28 states, SCN caused the greatest total dollar loss (~$800 USD/hectare) with a peak loss of just under $1600 USD/hectare in 2012. Additionally, over 30% of yield loss that occurs in SCN infested fields is without any noticeable aboveground symptoms [11]. A second study of about 15 years in more than 25,000 experimental plots at 122 location years highlighted that the number of virulent SCN populations reproducing on PI 88,788 grew from 2001–2015 due to the overuse of resistant cultivars. However, no effects were seen on Peking-derived varieties within the same study [12]. The study emphasized the critical need for novel sources of resistance as the usefulness of current resistance is continually diminishing [12]. Images of soybean roots infected with SCN are shown in Figure 1a,b in addition to a comparison of an SCN cyst vs. a nodule on roots in Figure 1b and the greenhouse facility for SCN phenotyping in Figure 1c.

### 1.3. Host–Pathogen Interactions

Plants have several lines of defense against pathogens. These include mechanical defences provided by, for example, the cuticle and cell wall. Plants also exhibit a first line of active plant defense by which pathogen-associated molecular patterns (PAMPs) induce pattern triggered immunity (PTI) [13]. Unfortunately, pathogens can supress various PTI components with effector proteins which they deliver into the plant. Plants also have a second actively induced immune system which is stronger, referred to as effector triggered immunity (ETI) [14]. ETI is much more specific, as it involves the recognition of these effectors/avirulence (*Avr*) genes by specific resistance (*R*) genes within the plant. It is known that PTI and ETI both take part in the innate immune response in plants, and in recent years growing evidence proposes that intricate interactions occur between pattern-recognition receptors in the PTI pathway and nucleotide-binding domain leucine-rich repeat containing receptors in the ETI pathway along with common signalling components which are shared by both [15]. Further research is required and the components that make them up are still largely unknown [16].

**Figure 2:**
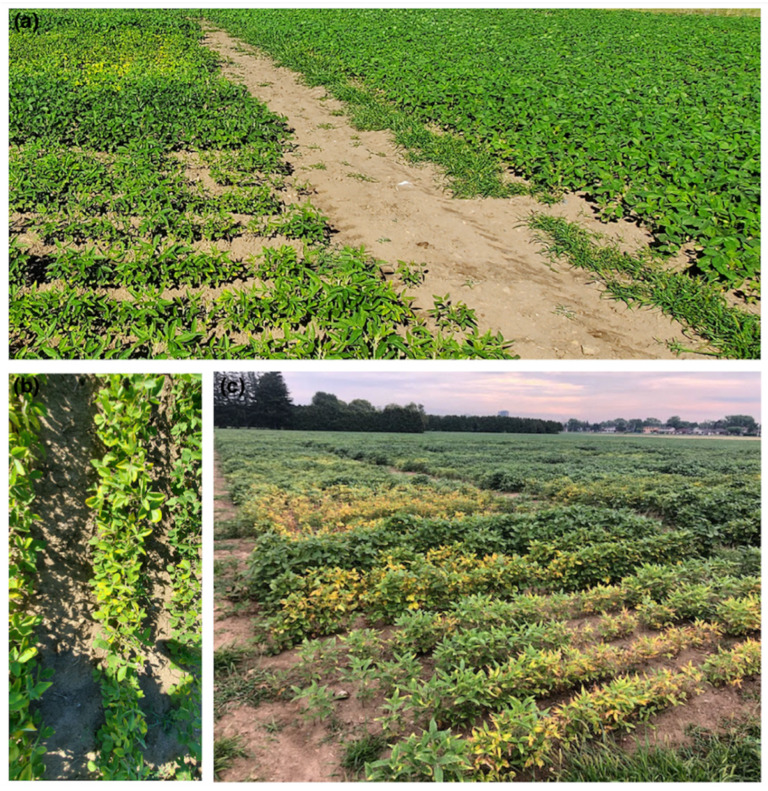
Soybean field in Agriculture and Agri-Food Canada, Ottawa Research and Development Centre (AAFC-ORDC) post SCN infection at different stages. (**a**) Soybean during early infection still appear relatively healthy; (**b**) leaf chlorosis and yellowing then becomes visible; (**c**) soybean plants become yellow and die during later stages of infection.

To date, only two soybean loci have been utilized on a large scale for SCN resistance: *Rhg1* and *Rhg4*. Specifically, SCN resistance is conferred by the recessive form of *Rhg1, rhg1* and the dominant form of *Rhg4*. The *Rhg1* locus was mapped to chromosome 18, with the *rhg1* gene itself displaying incomplete dominance. *Rhg4* was mapped to chromosome 8 [17,18,19,20]. *rhg1* is made up of a 31 kb multi-gene segment coding for three different proteins all involved in resistance [21]. The first is an α-SNAP protein (GmSNAP18), the second is a wound-inducible domain protein (WI12) (GmWI12) and the third is an amino acid transporter (AAT) (GmAAT) [21,22]. *Rhg-1* has two resistant alleles: *rhg1-a* (Peking-type) resistance with low copy number (3 or less copies of GmSNAP18, GmAAT, and GmWI12 in one genomic segment) and *rhg1-b* (PI 88788-type) resistance with a high copy number (4 or more copies of GmSNAP18, GmAAT and GmWI12 in one genomic segment) [23]. The *rhg1-a* allele carries a retrotransposon in the α-SNAP protein, while the α-SNAP protein in *rhg1-b* does not. This causes the *rhg1-a* “Peking-type” varieties to require *Rhg4* for complete resistance, while *rhg1-b* “PI 88788-type” varieties do not. *Rhg4* codes for a cytosolic serine hydroxymethyltransferase (SHMT) protein, which is responsible for resistance against SCN [20]. Having only two soybean resistance loci (*rhg1* and *Rhg4*) to SCN will not be sustainable for much longer, and resistance breakdown with more aggressive SCN populations are inevitable. Stages of SCN infection in fields are seen in Figure 2a,b, while later stages of infection are shown in Figure 2c.

### 1.4. Recent Work on Soybean Resistance against SCN

Because of the increasing importance of soybean across the world and the threats that SCN poses to soybean productivity, newly available tools have been employed. This is illustrated in recent review papers summarizing available resistance genes, molecular markers and new breeding strategies [24,25]. However, major progress has also been made in identifying novel genes and gene networks responsible for defense response regulation. Soybean–SCN interaction studies have largely been expanded within the last couple of years using a diversity of techniques including methylation studies, transgenics, large scale genomic, proteomic, transcriptomic, fine mapping/novel QTL identification and much more. These various research approaches have been used to study the soybean-SCN host–pathogen interactions and breeding strategies, as well as soybean’s response to SCN infection at the molecular and transcriptional level [25]. Much is still unknown, and challenges are being faced in developing long term effective strategies for controlling this nematode.

The primary objective of this article is to review the research done on the soybean-SCN relationship in the hopes of understanding the challenges we will likely encounter.

## 2. What Is New at the rhg1 and Rhg4 Loci?

The interaction of *rhg1* and *Rhg4* for resistance against SCN was clearly established in the past [26]. Since then, new findings have expanded our understanding of these systems.

### 2.1. α- SNAP

As a component of *rhg1*, the soybean gene GmSNAP18 codes for a soluble N-ethylmelaimide sensitive factor (NSF) attachment protein (α-SNAP) for which multiple haplotypes exist, each conferring a different type of resistance [22]. Using a positional cloning technique, along with region-specific extraction sequencing (RES-Seq) in resistant and susceptible lines, it was shown that Haplotype I (*rhg1-a*) carried the Peking-type resistance while Haplotype II (*rhg1-b*) carried PI 88788-type resistance and Haplotype III carried the susceptible version of GmSNAP18 (*rhg1-s*). The transcript levels of GmSNAP18 were 2.1 times higher in the *rhg1-a* resistant cultivars than the susceptible *rhg1-s* cultivars. They were also 8.3 times higher in the *rhg1-b* resistant cultivars than *rhg1-s* under uninfected conditions and even higher during infection. The *rhg1-a* allele also carries a retrotransposon in the α-SNAP protein while the α-SNAP protein in *rhg1-b* does not [23]. In mammalian genomes, the α-SNAP protein works with NSF which together act to mediate trafficking, disassembling and reusing of other important proteins associated with vesicle docking and fusion. NSF proteins are always encoded because null mutations are lethal in animals. Interestingly, soybean cultivars carrying the SCN-resistant *rhg1* haplotypes encode an unusual α-SNAP protein, which does not bind well with NSF, disrupting vesicle trafficking and leading to the death of the cell. However, a gene encoding a novel form of NSF protein, found on chromosome 7, had a unique N-domain that mitigated both toxicity and poor NSF binding of *rhg1* α-SNAPs during SCN resistance [27]. It was shown that resistant *rhg1* soybean contained the unique NSF_Chr07_ (termed NSF_RAN07_ for “Rhg1-associated NSF on chromosome 07”) while the susceptible ones contained the wild-type NSF_Chr07_.

The molecular mechanism of soybean’s resistance to SCN was further explored by a group that identified two syntaxins of the t-SNARE (SNAP REceptor) family that interact with the α-SNAP protein [28]. The authors used yeast-two-hybrid assays in addition to knockout methods to confirm the role of two syntaxin1 genes, Syn12 and Syn16, in SCN resistance [28]. The importance of syntaxin and the SNARE regulon was also explored through a homologue of the defense regulon found in *Arabidopsis thaliana* containing syntaxin PENETRATION1, an ATP-binding cassette and a secreted glucosidase [29]. Previous studies showed callose as being present during the defense process in plants against different pathosystems through a process involving vesicle membrane proteins and syntaxins [30]. The authors suggested that since myosin and SNARE components function in defense against SCN, then callose synthesis may also play a role. The results of their experiments in both overexpressing and knocking out callose genes confirmed the role of callose in defense. This study allowed an expansion of the already known central defense role and vesicle trafficking, adding callose synthase to factors responsible for defense against SCN. Another group also identified a SNARE protein interacting with α-SNAP (GmSYP31A) [31]. Transgenic hairy root soybean plants overexpressing GmSYP31A in susceptible Williams 82 led to increased resistance to SCN while RNAi silencing of GmSYP31A led to SCN susceptibility in resistant lines. Further analysis utilizing green fluorescent protein (GFP) revealed endoplasmic reticulum-Golgi trafficking and exocytosis defects with overexpressed GmSYP31. It was suggested that the interaction of the secretory protein GmSYP31A and the voltage-dependent anion channel played a role in the vesicle trafficking pathway as well as in mitochondrial-mediated cell death, which led to SCN resistance. This area of research was also explored by other researchers who demonstrated that the Conversed Oligomeric Golgi (COG) complex plays a role in retrograde trafficking of many proteins, including syntaxins, which interact with the NSF α-SNAP protein conferring SCN resistance [32]. Overexpression of 14 out of the 16 COG genes in susceptible soybean cultivar showed SCN suppression by 50% or more. Additionally, altered expression levels of the COG genes had an impact on transcript abundance of syntaxin 31, and its involvement in SCN resistance [32].

Membrane trafficking modifications in resistant reactions caused by α-SNAP-syntaxin and subsequent interactions with the COG complex also influence exocytosis. A paper was published identifying 61 exocyst genes, some of which were differentially expressed in the syncytium during the defense response to SCN [33]. The authors then further dove into 9 recently identified MAPK genes involved in resistance and their involvement with exocyst genes [34]. This study demonstrated the importance of the tethering stage of vesicle transport and its role in defense against SCN, and also demonstrated that exocyst genes are controlled by MAPK genes. The importance of MAPKs in signal transduction highlighted interactions with several defense genes, including a homolog of a pathogenesis-related 1 gene (PR1-6) that is induced by GmMAPK4-1, which may explain how the MAPK4-1 gene functions in defense [34]. RNA-seq analysis of transgenic soybean lines overexpressing the nine MAPKs involved in SCN defense led to the identification of several differentially expressed genes implicated in the resistance reaction [35]. From those, 71 were found to have transcripts in SCN syncytia in soybean roots, of which 45 had no expression prior to SCN infection. Eight proteins also had secretion signals, including glycosyl hydrolases, endomembrane protein, galactose mutarotase-like, pathogenesis-related thaumatin, FASCICLIN-like arabinogalactan protein and peroxidase. Functional validations confirmed the roles of some of these genes in defense. Xyloglucan endotransglycosylase/hydrolase (XTH) was also found to be highly expressed in syncytia and reduced infection when artificially overexpressed in a susceptible cultivar [36]. The protein functions in cutting and rejoining xyloglucan (XyG) chains to allow cell expansion. Further analysis into the mechanism of how this protein functions in resistance is necessary; however, the authors identified that increasing XTH42 leads to a decrease in XyG chain length, while the decrease in XTH43 transcripts increase XyG chain length, which is believed to have a role on the cell wall and its ability to expand and form a syncytium.

### 2.2. WI12

Recent findings within another component of *rhg1*, the wound-inducible domain protein (WI12) (GmWI12), suggested that the WI12_Rhg1_ protein interacts with DELLA proteins [37]. DELLA proteins are negative regulators of the gibberellic acid (GA) signalling pathway associated with the plant’s immune response and its survival [38,39]. The authors found that *WI12* knockout roots reduced *DELLA18* expression levels and that the two proteins directly interact based on yeast and plant experiments. A double knockout of *DELLA18* and its homolog *DELLA11* significantly increased the number of female nematodes on Peking roots. Finally, the authors also highlight the involvement of plant hormones GA, Jasmonic Acid (JA) and Salicylic Acid (SA), controlled by DELLA, in SCN resistance.

### 2.3. SHMT

The GmSHMT08 gene at *Rhg4* encodes a serine hydroxymethyltransferase (SHMT). The Peking-type lines (*rhg1-a*) are fully dependent on a specific allele of SHMT at *Rhg4* for SCN resistance [40]. A series of GmSHMT08 mutants obtained by forward genetics screening confirmed that Peking-type is mechanistically different from PI 88788-type resistance [40]. It is now established that the Peking-type, *rhg1-a* allele (low copy number) has higher resistance due to its interaction with *Rhg4* while the PI 88788-type *rhg1-b* allele (high copy number) does not benefit from the presence of *Rhg4* [41]. This was confirmed by deep re-sequencing of 106 soybean accessions which were also challenged with five different SCN HG types [42]. At least 5.6 *rhg1* copies were required for PI88788-type resistance which was independent of the *Rhg4* haplotype. However, due to the presence of a retrotransposon within the α-SNAP protein and copy number dropping below 5.6 (1.9–3.5), the *Rhg4* haplotype was necessary for resistance in Peking cultivars. SHMT catalyzes the conversion of L-serine to glycine and tetrahydrofolate to 5,10-methylenetetrahydrofolate. The resistant *Rhg4* allele differs from the susceptible by two polymorphisms [20]. The SHMT structure was compared by applying homology modelling between susceptible and resistant lines without observing major structural changes, although a slight rotation in the small domain of the susceptible enzyme was noted [43]. Near the entrance of the THF-binding site, this structure includes a loop which in the resistant line looks disordered. The effects of this disordered loop were tested and appeared to severely impair binding affinity for folate. This step is important because folate is essential for SCN’s development since the nematode is unable to synthesize it. These results indicated that SCN resistance in relation to *Rhg4* might be related to impairment in folate binding. However, the direct interaction of SHMT and α-SNAP was also proposed [44]. The products of both GmSHMT08 and GmSNAP18 were localized in the cytosol, supporting the hypothesis that the proteins could physically interact [44,45]. This interaction is thought to be facilitated by the presence of GmPR08-Bet VI, a pathogenesis-related protein (PR-10) which is known to bind to hormones, lipids and antibiotics which are bulky hydrophobic compounds [46]. Overexpression of this protein resulted in a 65% reduction in the number of cysts compared to control treatments. Bimolecular fluorescence complementation assays confirmed physical interactions between GmSHMT08 and GmPr08-Bet VI, which were enhanced when GmSNAP18 was also present. Interaction between *rhg1* and *Rhg4* in SCN resistance could therefore be the result of a multiprotein complex composed of GmSHMT08/GmSNAP18/GmPR08-Bet VI [47].

## 3. Defense Gene Activation and Epigenetic Control

The recognition of SCN by the plant in incompatible interactions will lead to drastic changes in gene expression. This transcriptomic modification appears to begin at a very early stage after infection (8 h post infection), during the migration phase of the J2 nematode to the vascular system and not only during the sedentary J3–J4 phase [48]. The defense response includes PTI related differentially expressed genes (DEGs), which became upregulated in resistant genotypes. In addition, nucleotide-binding site leucine-rich repeat (NBS-LRR) genes expression was also induced which meant that both PTI and ETI pathways become triggered within the first 8 h of infection. This shows that the soybean host begins defense and changes defense-related gene expression long before the nematode chooses a feeding site [48]. Expression of defense genes was also seen in the susceptible genotypes, but the reaction produced by the plant does not seem to be enough for resistance in susceptible cases. The differential response of susceptible and resistant soybean cultivars was notably studied using a metabolomics approach [49]. The authors identified 14 significantly differential expressed metabolites in the resistant cultivar that likely play a role in SCN defense.

Epigenetic variations in plants are known to influence a variety of traits and have been suggested to be included in crop breeding programs [50]. Recent work in soybean revealed that the methylome profile of resistant and susceptible lines (both control and infected) differ significantly [51]. The authors used near-isogenic lines (NILs) from a cross between susceptible and resistant soybean plants that differ at GmSHMT08. Methylation was reduced in response to SCN infection in the susceptible NILs, especially for protein-coding genes and transposable elements, while methylation increased in protein-coding genes and transposable elements for resistant NILs. Further analysis identified 112 and 1668 DEGs in resistant NILs and susceptible NILs, respectively. The functions affected in susceptible NILs were consistent with those usually modulated by cyst nematode effectors. Interestingly, heritable and novel non-parental differentially methylated regions were identified and shown to overlap with genes involved in soybean-SCN interactions [51]. In addition to the genes that were differentially methylated following SCN infection, the authors then identified several microRNA (miRNA) genes [52]. Four miRNA genes (gma-miR1520b, gma-miR5032, gma-miR5043, and gma-miR2107-ch16) showed opposing methylation patterns (hyper- vs, hypo-methylated) in susceptible NILs and resistant NILs. Transgenic hairy root lines for these miRNA genes confirmed their implication in resistance [52]. Overall, these studies highlighted the putative roles of miRNA in SCN resistance and how epigenetic mechanisms can regulate these processes.

## 4. Identifying Novel Sources of Resistance

Even if PI 88,788 and Peking resistance genes have dominated breeding programs, other sources of resistance, like PI 437654, are known to carry distinct resistance genes and will be crucial for the development of new breeding lines. Many new QTLs or resistance genes have been identified recently and some markers are available for marker-assisted selection. Most studies used recombinant inbred lines (RILs) from resistant × susceptible crosses and linkage maps to localize new resistance loci. Following these methods, new QTLs on Chr. 11 and on Chr. 08 were identified from RIL populations derived from A95-684043 × LS98-0582 and A95-684043 × LS94-3207, respectively (Table 1) [53]. Genome-wide association studies (GWAS) were also performed by different teams. One team genotyped 172 soybean lines and identified single nucleotide polymorphisms (SNPs) linked with chlorophyll content as the phenotype for tolerance to SCN [54]. Sixteen SNPs located on different chromosomes were found, of which most were in previously reported QTLs, except two on Chr. 03 and one on Chr. 06 that represent novel targets to diversify SCN resistance. This study was a first of its kind to focus on leaf chlorophyll content as a phenotype in order to identify new QTLs exhibiting resistance to SCN, compared to the usual evaluation based on SCN reproduction measured from cyst counts on infected soybean roots. Another GWAS compared 461 soybean lines from 28 different countries with varying levels of resistance [55]. Twelve important SNPs for SCN resistance were identified on chromosomes 7, 8, 10, and 18. The region found on Chr. 07 overlapped with a previous GWAS study with SCN resistance [56,57] but no other QTLs for SCN resistance were located on Chr. 10. This study therefore identified novel SCN resistance candidate genes for use by breeders in addition to *rhg1* and *Rhg4*. It suggested 24 genes potentially conferring SCN resistance with gene ontology pertaining to: LRR, cytochrome P450, DNA synthase, Ring/U-box, and transcription regulation. Researchers also identified a new resistance QTL on Chr. 07 [58]. A genetic linkage map of the RIL population from a cross between the resistant line PI 494,182 (Suzuhime), and the early maturity cultivar Costaud (MG 000) led to the identification of six significant QTLs (CSqSCN-1-6) correlated with SCN resistance. CSqSCN-1, 3 and 6 overlapped with the previously reported QTL’s for *GmSNAP18*, *GmSHMT08* and *GmSNAP11* genes respectively. However, an unreported locus, CSqSCN-4, was identified on Chr. 07, which appeared to contribute to resistance to a more virulent HG type (2.5.7). Overall, this study confirmed resistance to virulent SCN in early maturity germplasm and identified new markers for breeding. Another exciting study identified a novel QTL (LG O) in soybean cultivar Pingliang demonstrating a type of resistance distinct from Peking and PI 88,788 [59]. Pingliang carries the low copy *rhg1-a* allele along with the susceptible *Rhg4* haplotype which led the authors to believe that a major novel locus was responsible for its resistance to SCN. Linkage mapping using RILs from Magellan (susceptible) × Pingliang (resistant) uncovered a novel QTL on Chr. 10 (qSCN-PL10). Three novel genes were identified as valuable candidates (Glyma.10G197000, Glyma.10G195800 and Glyma.10G195900) and some of the metabolic processes that could be at play in producing a higher level of ROS responsible for immunity in Pingliang were highlighted. A fine-mapping study using PI 567516C, a line with broad-spectrum resistance to SCN, also confirmed a QTL (*qSCN10*) on Chr. 10 [60] that was previously identified [61,62]. The resistance in the cultivar carrying *qSCN10* was different from the typical PI88788 *rhg1* resistant cultivar as it contained three copies of *rhg1*, as Peking did, but not the *Rhg4* haplotype which is needed for resistance [45,63,64,65]. PI 567516C was challenged with multiple HG types and conferred moderate levels of resistance. Four candidate genes responsible for this resistance were identified and coded for a bZIP transcription factor, a receptor-like kinase, a sucrose non-fermenting family protein and a CC-NBS-LRR protein. Another novel QTL, located on Chr. 18 (*qSCN18*) was identified in the same cultivar (PI 567516C), which together with *qSCN10* conferred strong resistance [66]. Fine mapping identified a region of 166 kbp that contained 23 candidate genes, from which Glyma.18g244600 (AP2 domain transcription factor family) was the only one displaying differential expression in response to SCN infection. The authors then developed breeder-friendly genotyping assays as a fast and effective diagnostic tool for marker-assisted selection for this novel QTL. Three soybean germplasms with resistance derived from PI 567516C have been registered recently: JTN-5316, JTN-5416 and JTN-5516 [67]. The soybean line Dongnong L-204, a green seed coat cultivar, also received a lot of attention as a resistant cultivar. First, RNA-seq analysis identified DEGs potentially involved in resistance, including several transcription factors [68]. Then, the same cultivar was challenged with a virulent SCN population (HG type 1.2.3.5.7) which confirmed the implication of several genes including GmRSCN4-1 and GmRSCN4-2 [69]. The two genes, which were overexpressed using a hairy root transformation in the susceptible cultivar Heinong 37, led to a substantial reduction in the number of developing SCN females.

## 5. Wild Soybean as a Resistance Reservoir

Soybean’s wild relative, *G. soja*, has been studied mainly to understand soybean domestication, but its high genetic diversity is known to contain desirable traits for crop improvement, including SCN resistance [70]. GWAS was conducted on 1032 *Glycine soja* accessions in order to have a better understanding of wild soybean resistance against SCN [71]. Ten SNPs significantly associated with resistance to SCN were found on chromosomes 2, 4, 9, 16 and 18, three of which were previously identified, but none of which were among the *rhg1* or *Rhg4* QTLs. These regions contained 83 gene models, and some were compatible with plant resistance against disease including: calcium-dependent phospholipid-binding protein, NB-ARC domains containing protein, LRR protein, cytochrome P450, and ethylene-responsive element binding factor. One specific gene, Glyma.18G102600, an NB-ARC domain containing protein, located in a strong linkage disequilibrium block on Chr. 18 seemed highly promising. A transcriptomics database of the response of resistant and susceptible *G. soja* accessions to SCN was also created [72]. Another GWAS on *G. soja* lines identified SNPs on chromosomes 18 and 19 as being significantly associated with resistance to SCN (HG 2.5.7), as well as identified 58 gene candidates [73]. From these, 16 were related to disease resistance, encoding LRR proteins, ring/U-box, receptor-like protein, and MYB transcription factor. Other authors compared transcript expression of the resistant *G. soja* line NRS100 to the well-known *G. max* Williams 82 (susceptible) and Peking (resistant). The resistant *G. soja* (NRS100) did not show any significant differential expression at SHMT, SNAP paralog or SNAP18 which are found in *rhg1* and *Rhg4*. The proposed defense mechanism in NRS100 included reduced JA signalling which allowed SA signals to induce a defense response, along with increased polyamine metabolism triggering H_2_O_2_ regulation and induction of PR proteins which defend the integrity of the cell walls and hinder pathogen invasion [74]. Finally, a cross between *G. max* and *G. soja* along with chromosome segment substitution lines (CSSLs) for QTL mapping of SCN resistance was performed [75]. Thirty-three QTLs were detected on 18 different chromosomes with high significance in relation to SCN resistance. The CSSLs combining positive alleles were highly resistant to SCN in absence of *rhg1* and *Rhg4.* These studies shed light on the importance of *G. soja* germplasm and new strategies for resistance breeding.

## 6. Novel Resistance Strategies and Breeding Approaches

Several studies have demonstrated the potential of transgenic lines to reach a solid level of resistance against SCN. One group has shown that the overexpression of a salicylic acid methyl transferase in a soybean hairy root system was highly detrimental to SCN [76]. RNA interference targeting reproduction and fitness genes in SCN was also used to demonstrate that host-derived gene silencing could be an effective strategy to improve resistance [77]. The availability of new gene editing systems such as clustered regularly interspaced short-palindromic repeats (CRISPR)-Cas9 should facilitate the development of such lines. This technology was recently used to engineer multiple traits in corn with the transgenes grouped in a single complex trait locus [78]. This opens interesting possibilities for gene pyramiding and trait stacking in soybean in order to reach a reliable level of resistance. In soybean, a CRISPR-Cas9 genome editing platform was developed to overcome some of the challenges associated with this technology and should facilitate future work [79].

Recently, BASF released a study in which they showed that the use of transgenic lines expressing the Cry14Ab protein has exceptional potential for controlling plant-parasitic nematodes [80]. They utilized the *Bacillus thuringiensis* delta-endotoxin, Cry14Ab for SCN control. In addition to interfering with feeding site establishment of the nematode, the Cry14Ab protein also stopped the development of juveniles. The reduction of cyst numbers was observed using different HG types (1.2.3.5.6 and 2.5.7) and was validated in the field. It was suggested that the protein damages the nematodes intestines, a mechanism similar to the toxins produced by *Bacillus thuringiensis* (*Bt)* crops to control insects.

Non-host resistance (NHR), the complex defense mechanisms that confer plant immunity [81], have also received some attention for soybean breeding. NHR traits are highly complex, yet it has been suggested that they could possibly be transferred into different species [82]. This was tested by a team that utilized an NHR gene from Arabidopsis to transform a susceptible soybean cultivar such as Williams 82 into one that is resistant against SCN [83]. The PSS30 gene encoding a folate transporter, *AtFOLT1,* was found to confer immunity against *Fusarium virguliforme* and *Phytophthora sojae* as folate levels rise due to infection in Arabidopsis. In contrast, the reduction of folate levels in mutants led to the loss of non-host immunity in *P. sojae* [82]. The authors overexpressed this gene in soybean through transgenic cultivar Williams 82. They observed a folate increase of 12% in transgenic infected soybean line vs. the non-transgenic infected line leading to enhanced resistance against SCN. This study proposed that folate is a key part of plants non-host immunity and that expression of NHR genes could be employed to build broad-spectrum resistance in crops. A transcriptomic analysis also explored the NHR of the soybean cultivar Lee (usually susceptible to SCN) to a divergent type of SCN named SCN_T_ (reproducing on tobacco) [84]. The study identified 3746 DEGs when cultivar Lee was challenged by SCN_T_ compared to only 602 with a standard SCN population. A single gene, coding for a peroxidase, was found to be upregulated in susceptible and downregulated in resistant interactions (Glyma06g15030). Most of the DEGs were associated with oxidoreductase activity.

It was suggested that inoculation of soybean with plant growth-promoting rhizobacteria (PGPR) could improve its resistance to SCN [85]. A follow-up study using transcriptomics and metabolomics was performed to explore the mechanisms leading to a 73% decrease in SCN number after inoculation with a *Bacillus simplex* strain [86]. The authors have identified several DEGs and distinct metabolomics profiles between inoculated and control plants. Based on the metabolite analysis it was shown that L-methionine, 4-vinylphenol, piperine as well as palmitic acid had higher concentrations in the inoculated plants infected by SCN. These molecules were tested for effectiveness as nematicidal compounds and resulted in high nematode mortality. Therefore, the authors proposed the use of PGPRs to regulate nematicidal metabolites’ gene expression in system-oriented management strategies.

Finally, one of the steps complicating the selection of resistant lines or the evaluation of management strategies is the phenotyping process. The precise determination of the level of resistance to SCN can only be achieved by counting the number of females developing on the roots. This is usually achieved by extracting the cysts from soil or roots using sieving or elutriation and counting them by visual enumeration under the microscope, which is extremely time consuming. Recently, new techniques were developed to improve soil extraction and automate egg or cyst enumeration. One of these methods uses centrifugation in a density gradient medium to purify SCN eggs before acquiring images from a high-resolution scanner or videos from a microfluidic system and automated counting with a deep-learning pipeline [87]. These algorithms based on convolutional networks were shown to be comparable to human evaluation in most situations [88]. The soil extraction and grinding of cysts (to release the eggs) were also automated in a robotic instrument that reproduced each step of a manual wet-sieving extraction [89]. Egg viability assessment can also be automated, for example, using a Complex Object Parametric Analyzer and Sorter (COPAS) system [90]. All of these developments will facilitate and accelerate the selection of resistant cultivars and the management of SCN.

## 7. Conclusions

Overall, the research listed above attempts to identify novel genes/proteins involved in SCN resistance and to improve our understanding of the interactions between soybean and SCN. SCN is spreading into many areas of the world as well as overcoming the existing resistance sources, meaning researchers must develop new methods to fight this pest. The review was written to summarize different types of work that have been conducted, and to identify research gaps, in hopes of taking a direction that would yield new and interesting results.

SCN resistance in soybean is mediated by *Rhg* genes and even though improvements have been made to identify novel genes involved in SCN resistance, this area of research remains largely under-studied. Advancements in sequencing techniques have made soybean and SCN genomes available which will speed our ability to identify specific SCN effectors as well as soybean-resistant components [91]. Additional innovations could also be made by pyramiding genes from a variety of different sources of resistance [24].

SCN’s effector proteins’ interaction with soybean resistance (*R*) proteins produce an interaction and metabolic pathway that is not yet clearly understood. Epigenetics and the switching of genes on and off through non-coding small RNA (sRNA), DNA methylation, mRNA, miRNA and histone modification is another area of research that is extremely significant and needs to be further explored [92]. This area of research is thought to be crucial in further understanding transcriptional gene silencing and the parasitism of this nematode [93].

In addition to soybean -omics research that is being conducted, recently, there has also been a shift to understanding and uncovering the soybean cyst nematode genome through chromosomal assembly [8], as well as genomic profiling of virulence genes in SCN [94]. It is believed that studying the nematode itself and learning more about its genome and protein interactions with soybean is also a valuable resource and will help us further expose novel genes and methods to overcome SCN in the fields. Finally, to develop markers for assisted breeding selection to quickly rise above SCN’s negative effects.

## Figures and Tables

**Table 1:** Summary of novel sources of resistance QTL’s and SNPs from Section 4 of the review. Regions in base pairs (bp) are based on Wm82.a2.v1. For a more up to date and complete list of QTL’s and SNPs please refer to SoyBase https://www.soybase.org (21 January 2022).

Population/Study	QTL/SNP	Chromosome	Markers, Regions and/or SNPs	Ref.
AX19286	*SCN-3*	08	Satt470–Satt228/116.7–154.1 (cM)	[53]
AX19287	*SCN-5*	11	Satt638–Satt197/37.7–46.4 (cM)	[53]
GWAS	*Gm03_3,334, 303_C_A*	03	3,334,303 (bp)	[54]
GWAS	*Gm03_39,574, 966_T_C*	03	39,574,966 (bp)	[54]
GWAS	*Gm06_50,593, 128_T_G*	06	50,593,128 (bp)	[54]
GWAS	*ss715606985*	10	40,672,699 (bp)	[55]
PI 494182	*CSqSCN-4*	07	19.8–22.9(cM)	[58]
Pingliang xiaoheidou	*qSCN-PL10*	10	Marker1015405–Marker1014475	[59]
PI 567516C	*qSCN10*	10	42,430,713–42,809,800 (bp)	[60]
PI 567516C	*qSCN18*	18	53,086,270–53,635,461 (bp)	[66]

## Data Availability

Not applicable.
